# ITGAM is a critical gene in ischemic stroke

**DOI:** 10.18632/aging.205729

**Published:** 2024-04-17

**Authors:** Lei Hou, Zhongchen Li, Xiaoli Guo, Jiatao Lv, Zonglei Chong, Yilei Xiao, Liyong Zhang, Zefu Li

**Affiliations:** 1 Department of Neurosurgery, Qilu Hospital of Shandong University, Jinan 250012, Shandong Province, P.R. China; 2 Department of Neurosurgery, Liaocheng People’s Hospital, Shandong Provincial Hospital, Cheeloo College of Medicine, Shandong University, Liaocheng 252000, Shandong Province, P.R. China; 3 Department of Pediatrics, Liaocheng People’s Hospital, Shandong Provincial Hospital, Cheeloo College of Medicine, Shandong University, Liaocheng 252000, Shandong Province, P.R. China; 4 Department of Neurosurgery, Binzhou Medical University Hospital, Binzhou Medical University, Binzhou 256603, Shandong Province, P.R. China

**Keywords:** enrichment analysis, hub gene, bioinformatics analysis, ROC

## Abstract

Background: Globally, ischemic stroke (IS) is ranked as the second most prevailing cause of mortality and is considered lethal to human health. This study aimed to identify genes and pathways involved in the onset and progression of IS.

Methods: GSE16561 and GSE22255 were downloaded from the Gene Expression Omnibus (GEO) database, merged, and subjected to batch effect removal using the ComBat method. The limma package was employed to identify the differentially expressed genes (DEGs), followed by enrichment analysis and protein-protein interaction (PPI) network construction. Afterward, the cytoHubba plugin was utilized to screen the hub genes. Finally, a ROC curve was generated to investigate the diagnostic value of hub genes. Validation analysis through a series of experiments including qPCR, Western blotting, TUNEL, and flow cytometry was performed.

Results: The analysis incorporated 59 IS samples and 44 control samples, revealing 226 DEGs, of which 152 were up-regulated and 74 were down-regulated. These DEGs were revealed to be linked with the inflammatory and immune responses through enrichment analyses. Overall, the ROC analysis revealed the remarkable diagnostic potential of ITGAM and MMP9 for IS. Quantitative assessment of these genes showed significant overexpression in IS patients. ITGAM modulation influenced the secretion of critical inflammatory cytokines, such as IL-1β, IL-6, and TNF-α, and had a distinct impact on neuronal apoptosis.

Conclusions: The inflammation and immune response were identified as potential pathological mechanisms of IS by bioinformatics and experiments. In addition, ITGAM may be considered a potential therapeutic target for IS.

## INTRODUCTION

Ischemic stroke (IS), a cerebrovascular disease with significant implications for human health, is a leading cause of mortality and long-term disability [1]. According to 2019 statistics, there were an estimated 12.2 million incident strokes globally (95% UI 11.0 million to 13.6 million), of which 62.4% were ischemic strokes (IS) [2]. The occurrence of IS at a younger age, along with associated complications, presents a substantial medical burden on society [3]. IS typically results from cellular death due to reduced local cerebral blood flow (CBF), leading to the development of an infarct core in ischemic brain tissue and distinct penumbral regions [4]. Despite advancements in research, the precise molecular mechanisms underlying IS remain poorly understood.

The previously published studies on the screening of genetic alterations at the genomic level have been further extended using microarray and bioinformatics analyses [5]. The molecular complexity of non-small cell lung cancer (NSCLC) has been illustrated utilizing microarray analysis, particularly the resistance to tyrosine kinase inhibitors, through the identification of biological processes, potential NSCLC targets, and gene expression signatures [6]. Furthermore, integrated transcriptomic and proteomic analyses and a thorough examination of biological networks detected the toxicological targets and networks utilized for the regulation of CCl4 in liver fibrosis [7]. Therefore, for the efficient treatment and accurate diagnosis of IS, it is necessary to explore a bioinformatics-based approach to elucidate the mechanisms of IS and study the prognosis-linked indicators as well as potential biomarkers.

This study conducted a comparative analysis of blood samples from individuals with ischemic stroke (IS) and normal individuals to identify differentially expressed genes (DEGs), which were subsequently analyzed by integrating two mRNA microarray datasets. Additionally, functional clustering analysis of DEGs was conducted, and a protein-protein interaction (PPI) network was constructed to identify hub genes. The potential drugs that could interact with these hub genes were predicted. Finally, the hub genes were validated through a series of experimental analyses including TUNEL, flow cytometry, quantitative polymerase chain reaction (qPCR), and Western blotting. Overall, the findings of this research add to the existing knowledge about the pathological mechanisms of IS at the fundamental molecular level.

## RESULTS

### Batch effect removal

**Figure 1:**
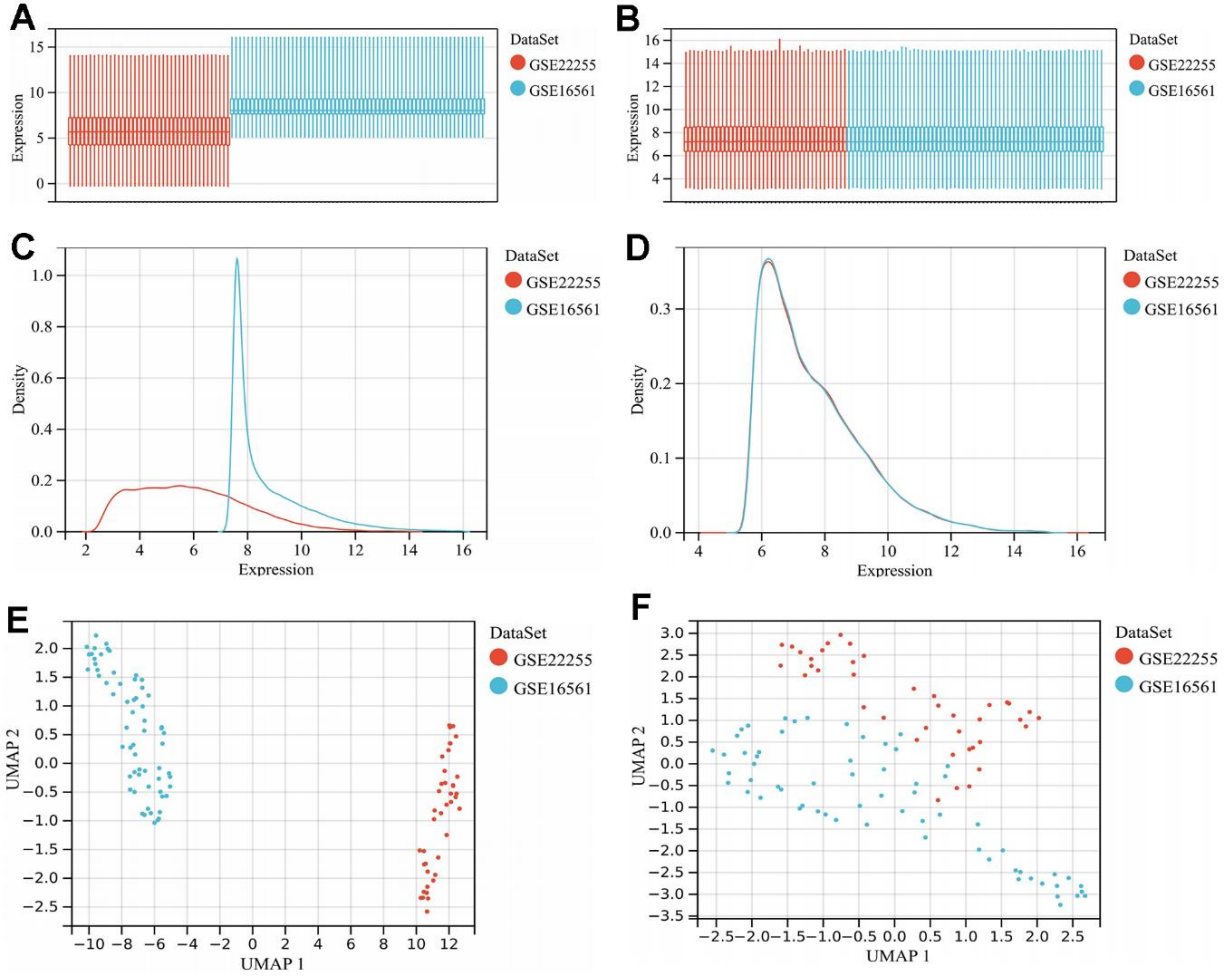
**Merging of GSE16561 and GSE22255 datasets and batch effect removal.** (**A**) Gene expression levels of the datasets before batch effect removal; (**B**) Gene expression levels of the merged dataset after batch effect removal; (**C**) Density plot of the datasets before batch effect removal; (**D**) Density plot of the merged dataset after batch effect removal; (**E**) UMAP plot of the datasets before batch effect removal; (**F**) UMAP plot of the merged dataset after batch effect removal. Abbreviations: UMAP, uniform manifold approximation and projection.

The box plot shows the large difference in sample distribution between the GSE22255 and GSE16561 datasets before batch effect removal (Figure 1A), suggesting a significant batch effect. However, the data distribution became consistent between the datasets, with their median in one line after removing the batch effect (Figure 1B). In addition, the density plot suggests a large difference in the sample distribution between the data sets before batch effect removal (Figure 1C). After removing the batch effect, the data distribution among the data sets tends to be consistent, and the mean and variance are similar (Figure 1D). In addition, the uniform manifold approximation and projection (UMAP) plot highlights the clustered distribution pattern of samples within each dataset before batch effect removal (Figure 1E). However, the samples from each dataset cluster intertwined with each other after the removal of the batch effect, suggesting a good removal (Figure 1F).

### DEG screening

**Figure 2:**
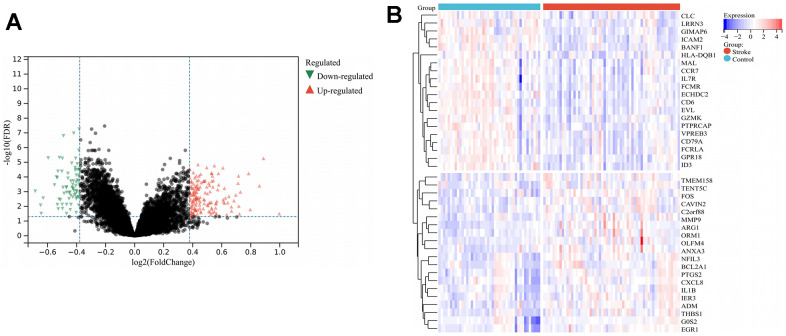
**DEGs screening.** (**A**) Volcano plot of IS-related DEGs, where the horizontal coordinate is log2FoldChange, the vertical coordinate is -log10(FDR), red triangles indicate up-regulated DEGs, green triangles indicate down-regulated DEGs and gray nodes indicate genes with no significant differential expression; (**B**) Heat map of 40 DEGs, where light-red represents disease samples, light-blue represents normal control samples, red represents high gene expression, and blue represents low gene expression. Abbreviations: IS, ischemic stroke; DEGs, differentially expressed genes; FDR, false discovery rate.

The DEGs were identified from the merged dataset of GSE22255 and GSE16561 using fold change > 1.3, and the adjusted P-value < 0.05 was utilized as the criteria for the screening of these genes. Finally, 226 DEGs comprising 152 up-regulated and 74 down-regulated genes were obtained (Figure 2A and Supplementary Table 1), and the identified up-regulated and down-regulated genes placed at the top twenty positions were visualized by the heat map (Figure 2B).

### Enrichment analyses

**Figure 3:**
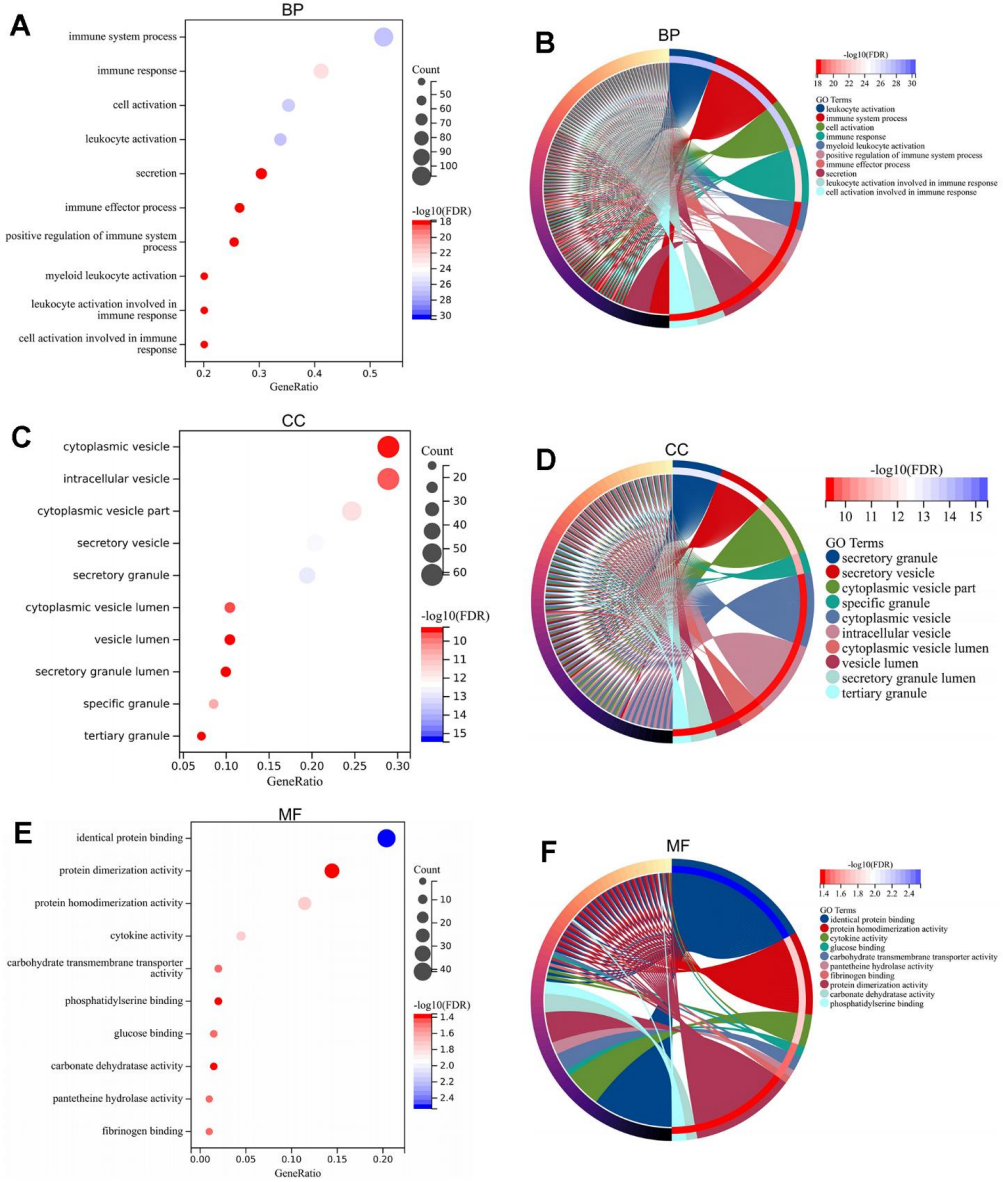
**GO enrichment analysis of DEGs.** GO-BP analysis for DEGs revealing significant terms via bubble plot (**A**) and linked genes by cnetplot (**B**). GO-CC analysis for DEGs revealing significant terms via bubble plot (**C**) and linked genes by cnetplot (**D**). GO-MF analysis for DEGs revealing significant terms via bubble plot (**E**) and linked genes by cnetplot (**F**). Abbreviations: DEGs, differentially expressed gene; GO, Gene Ontology; BP, biological process; CC, cellular component; MF, molecular function.

**Figure 4:**
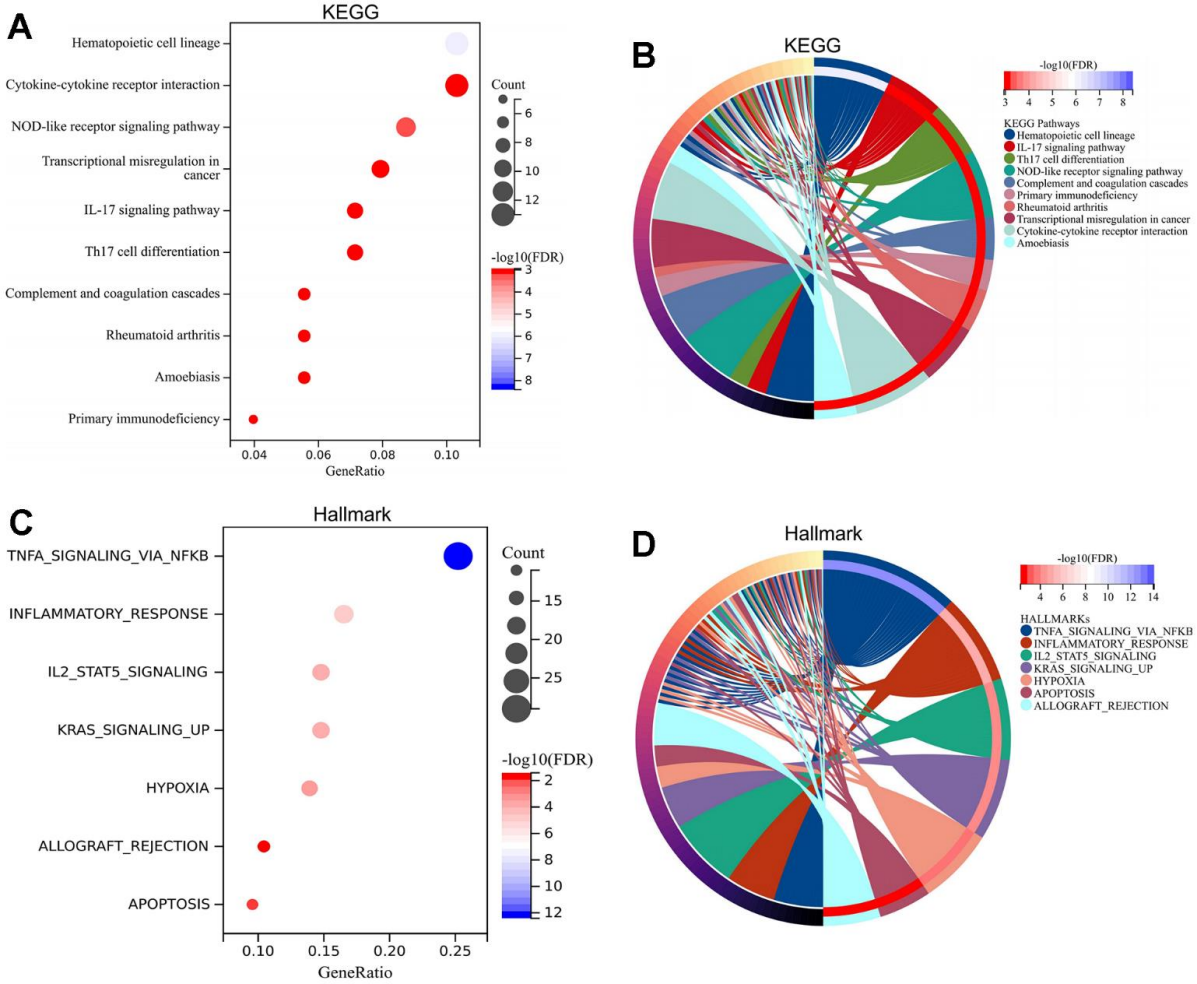
**Enrichment analysis of pathways for DEGs.** KEGG analysis for DEGs revealing significant pathways via bubble plot (**A**) and linked genes by cnetplot (**B**). Hallmark analysis for DEGs revealing significant pathways via bubble plot (**C**) and linked genes by cnetplot (**D**). Abbreviations: DEGs, differentially expressed gene; KEGG, Kyoto Encyclopedia of Genes and Genomes.

The associated biological functions of the DEGs were analyzed through enrichment analysis of the GO and KEGG. The enriched GO terms included immune system process, immune response, cytoplasmic vesicle, intracellular vesicle, identical protein binding, protein dimerization activity, and other terms (Figure 3A, 3C, 3E). In addition, the enriched genes for specific GO terms are displayed by cnetplots (Figure 3B, 3D, 3F). The enriched KEGG pathway included hematopoietic cell lineage, NOD-like receptor signaling pathway, TNFA signaling via NFKB, inflammatory response, cytokine–cytokine receptor interaction, and other functions (Figure 4A, 4C). Afterward, the enriched genes for specific KEGG pathways were mapped (Figure 4B, 4D).

### PPI network construction and hub gene screening

**Figure 5:**
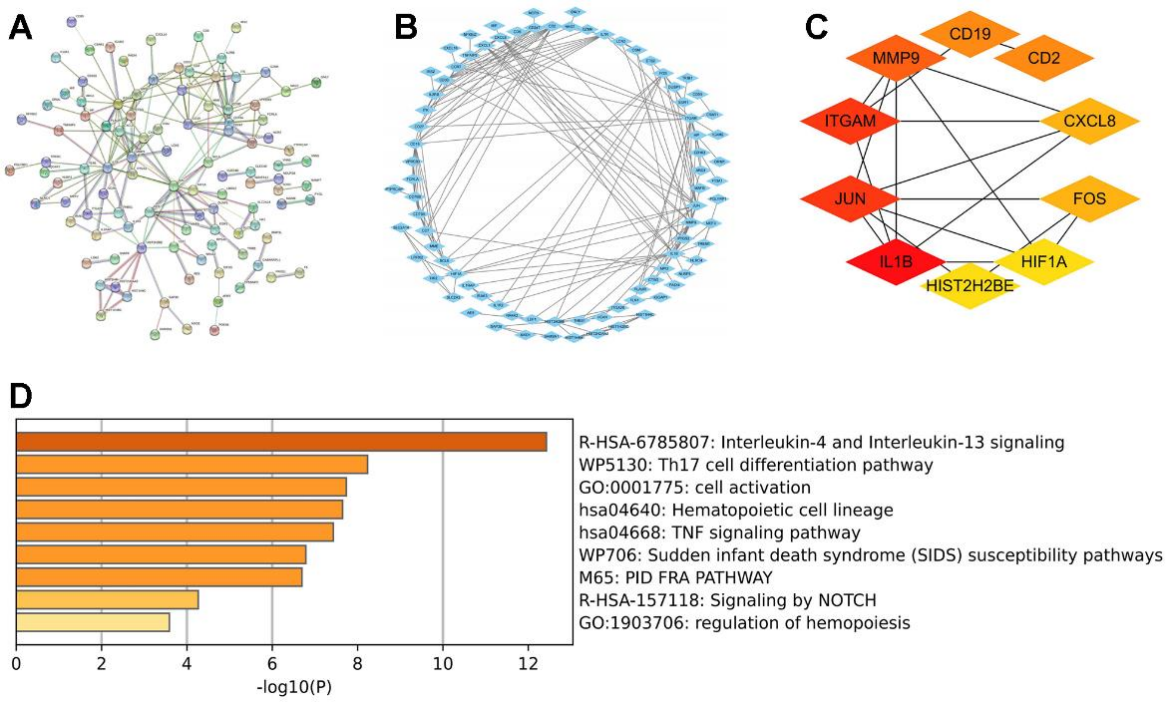
**Hub gene analysis.** (**A**) Construction of PPI network for DEGs through STRING database. (**B**) The PPI network was optimized by Cytoscape software. (**C**) Ten hub genes were filtered out per the degree method utilizing the Cytoscape’s cytoHubba plugin. (**D**) Enrichment analysis of 10 hub genes was performed by Metascape database. Abbreviations: PPI, protein-protein interaction; DEGs, differentially expressed genes.

The interactions between DEGs were examined and studied by fabricating and then optimizing a PPI network through the STRING database (Figure 5A) and the Cytoscape (Figure 5B), respectively. Afterward, ten hub genes were filtered out per the degree method utilizing Cytoscape’s cytoHubba plugin (Figure 5C) whose enrichment analyses depicted their enrichment mainly in interleukin-4 and interleukin-13 signaling, Th17 cell differentiation pathway, cell activation, and other processes (Figure 5D).

### ROC analysis for diagnosis

**Figure 6:**
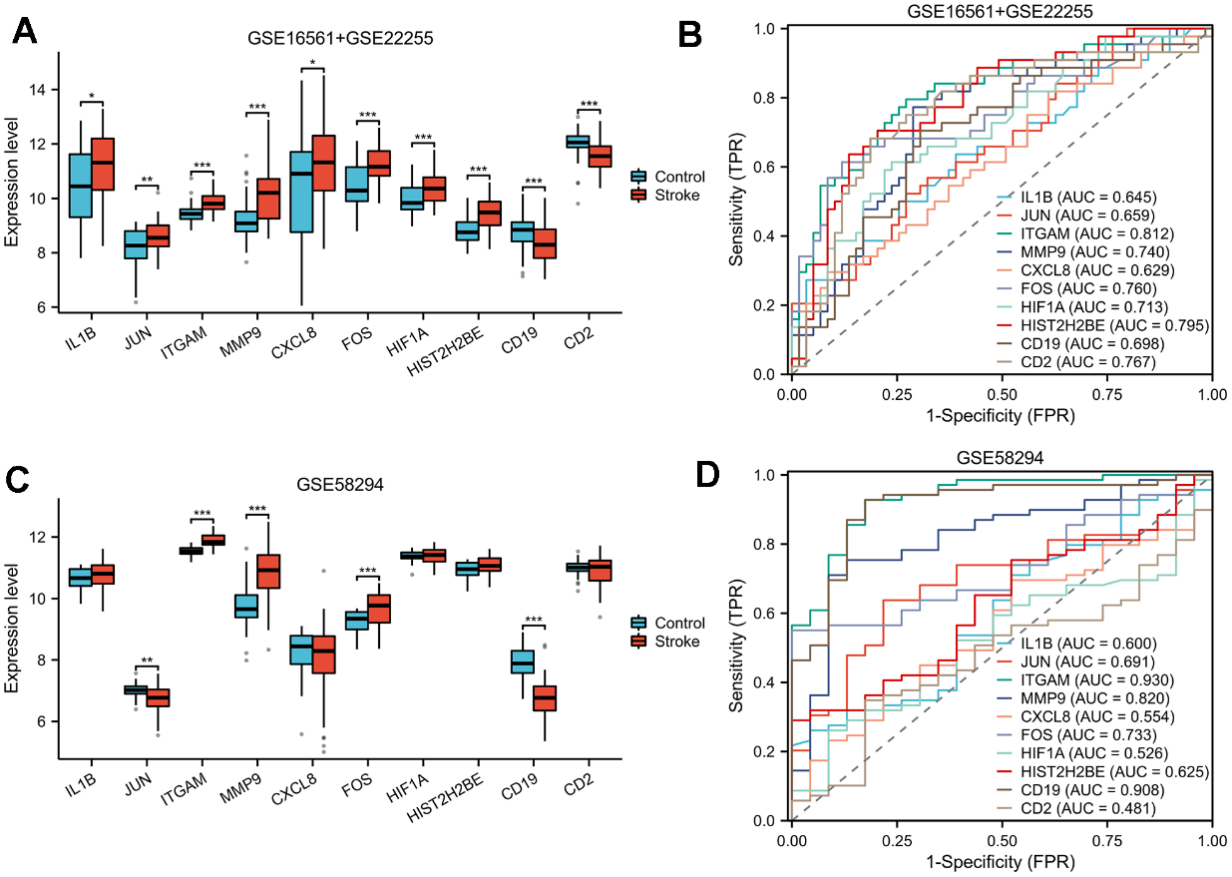
**ROC analysis.** In both GSE16561+GSE22255 (**A**) and GSE58294 (**C**) datasets, box plots were used to illustrate the expression patterns of hub genes between IS and normal samples. To determine the diagnostic performance of hub genes, we conducted ROC analysis for the GSE16561+GSE22255 (**B**) and GSE58294 (**D**) datasets. ROC, receiver operating characteristic; AUC, area under curve; FPR, false positive rate; TPR, true positive rate.

The box plot illustrated the expression profile of identified hub genes in IS and normal samples. The majority of hub genes were upregulated in IS tissues compared to normal tissues in the GSE16561+GSE22255 dataset, except for CD19 and CD2, which were downregulated (Figure 6A). In the GSE58294 dataset, half of the hub genes were found to be differentially expressed (Figure 6C). Our analysis revealed that the expression levels of the majority of hub genes were comparable among IS patients of different ages and genders, with insignificant differences observed (Supplementary Figure 1).

Our ROC analysis of the GSE16561+GSE22255 dataset demonstrated a remarkable diagnostic potential for ITGAM (AUC = 0.812), HIST2H2BE (AUC = 0.795), CD2 (AUC = 0.767) and MMP9 (AUC = 0.740) in distinguishing between normal and IS groups (Figure 6B). Our analysis of the GSE58294 dataset revealed remarkable diagnostic potential for ITGAM (AUC =0.930), CD19 (AUC=0.908) and MMP9 (AUC=0.820) in accurately differentiating between normal and IS groups, as indicated by the ROC curves (Figure 6D).

### TF, miRNA, and drug predictions

The TFs, miRNAs, and drugs acting on ten hub genes were predicted by the Enrichr platform. The TRRUST database transcriptional predictions suggested that the key TFs were NFKBIA, ARNT, and STAT3 (Table 1). In addition, the miRTarBase predictions identified the key miRNAs to be hsa-miR-338-3p, hsa-miR-5089-3p, and hsa-miR-155-5p (Table 2). According to the DSigDB database search, the key drugs were SB202190, 1,9-Pyrazoloanthrone, and chitosamine (Table 3). These TFs and miRNAs may have a potentially crucial role in the progression of IS, and the predicted drugs could potentially be used as drugs for the treatment of IS.

**Table 1:** Prediction of transcription factors regulating hub genes.

**Term**	**P-value**	**Combined score**	**Genes**
NFKBIA	8.68E-08	8698.922199	CXCL8;IL1B;MMP9
ARNT	1.02E-07	8105.110307	JUN;FOS;HIF1A
STAT3	4.95E-07	1392.434933	CXCL8;FOS;MMP9;HIF1A
JUN	6.00E-07	1307.154489	JUN;CXCL8;IL1B;MMP9
SIRT1	1.54E-06	2542.462924	IL1B;MMP9;HIF1A
FOS	2.60E-06	2034.951617	CXCL8;FOS;MMP9
IKBKB	3.37E-06	15739.23473	CXCL8;MMP9
HSF2	4.72E-06	12254.80045	FOS;HIF1A
HDAC1	5.05E-06	1531.250017	CXCL8;FOS;MMP9
ZFP36	6.29E-06	9972.498783	CXCL8;HIF1A

**Table 2:** Prediction of miRNAs regulating hub gene.

**Term**	**P-value**	**Combined score**	**Genes**
hsa-miR-338-3p	4.05E-05	618.9836824	FOS;MMP9;HIF1A
hsa-miR-5089-3p	2.10E-04	1005.249888	FOS;HIF1A
hsa-miR-155-5p	6.97E-04	102.9123975	JUN;CXCL8;FOS;HIF1A
hsa-miR-204-5p	8.52E-04	149.8799815	CXCL8;IL1B;MMP9
hsa-miR-139-5p	0.001195235	324.8248081	JUN;FOS
hsa-miR-429	0.002448892	200.1450746	JUN;HIF1A
hsa-miR-21-5p	0.002899587	79.83034502	IL1B;MMP9;HIF1A
hsa-miR-3622b-5p	0.003126287	169.1208984	CD19;FOS
hsa-miR-146a-5p	0.004288773	135.5473298	CXCL8;FOS
hsa-miR-106a-5p	0.004505057	62.78231133	CXCL8;IL1B;HIF1A

**Table 3:** Prediction of drugs acting on hub genes.

**Term**	**P-value**	**Combined score**	**Genes**
SB 202190 CTD 00003161	3.78E-15	18362.12229	JUN;ITGAM;CXCL8;IL1B;FOS;MMP9;HIF1A
1,9-Pyrazoloanthrone CTD 00003948	5.19E-13	7516.793025	JUN;ITGAM;CXCL8;IL1B;FOS;MMP9;HIF1A
chitosamine CTD 00006030	1.10E-12	10280.78243	JUN;CXCL8;IL1B;FOS;MMP9;HIF1A
TPEN CTD 00001994	5.54E-12	4853.883587	JUN;ITGAM;CXCL8;IL1B;MMP9;HIF1A;HIST2H2BE
PD 98059 CTD 00003206	7.09E-12	4635.940789	JUN;ITGAM;CXCL8;IL1B;FOS;MMP9;HIF1A
STYRENE CTD 00001125	1.02E-11	12947.91105	JUN;ITGAM;CXCL8;IL1B;FOS
Acetovanillone CTD 00002374	1.02E-11	12947.91105	JUN;ITGAM;CXCL8;IL1B;HIF1A
6401-97-4 CTD 00000925	1.04E-11	48133.11297	JUN;CXCL8;FOS;MMP9
139890-68-9 CTD 00002746	1.15E-11	12564.93891	JUN;CXCL8;IL1B;FOS;MMP9
sulfasalazine CTD 00006719	4.68E-11	8781.190432	ITGAM;CXCL8;IL1B;MMP9;HIF1A

### Differential expression of MMP9 and ITGAM in ischemic stroke

**Figure 7:**
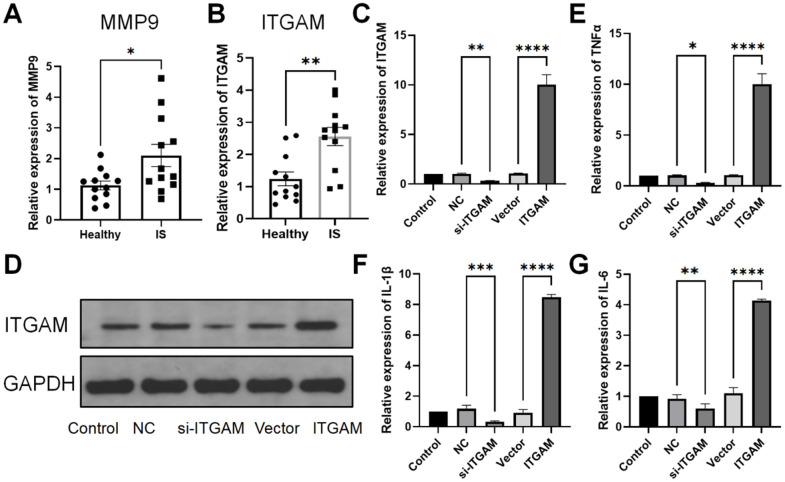
**qPCR and Western blot.** Relative expression analysis of MMP9 and ITGAM in IS and normal patients. (**A**, **B**) represent the relative expression of MMP9 and ITGAM mRNA in control and IS patients by qPCR. (**C**) q-PCR relative expression of ITGAM in different settings. (**D**) Western blot of ITGAM and GAPDH in different groups. (**E**–**G**) Relative expression of mRNA of TNF-α, IL-1β, and IL-6 when interfering with ITGAM in ischemic stroke. *P < 0.05, **P < 0.01 (n = 4 experiments, Student’s t-test).

The expression levels of both MMP9 and ITGAM were quantitatively assessed in the ischemic stroke (IS) and control groups. Comparative analysis revealed that the relative expression level of ITGAM was notably higher than that of MMP9 in the IS group, compared to controls (Figure 7A, 7B). Given the substantial upregulation of ITGAM, we chose to focus on this gene for subsequent targeted interventions.

Subsequently, the feasibility of modulating ITGAM levels was assessed through targeted interventions, specifically employing siRNA for gene silencing (si-ITGAM) and a specific construct for gene overexpression (ITGAM). The findings revealed that the interference efficiency of si-ITGAM was quantified as 0.325 and the overexpression efficiency as 10.019 through qPCR. The control interventions (NC and Vector groups) showed no significant alterations, demonstrating the specificity of the interventions (Figure 7C).

Western blot analysis substantiated these findings, with protein expression levels of ITGAM relative to GAPDH being consistent with the qPCR data. These experiments unequivocally verify that ITGAM can be selectively silenced or overexpressed, offering a viable path for targeted therapeutic intervention (Figure 7D).

We further investigated the influence of ITGAM on the release of inflammatory cytokines. Interfering with ITGAM was found to stimulate the secretion of critical inflammatory markers, including IL-1β, IL-6, and TNF-α. In contrast, the overexpression of ITGAM led to the suppression of these inflammatory mediators (Figure 7E–7G).

### Impact of ITGAM modulation on neuronal apoptosis: TUNEL and flow cytometry analyses

**Figure 8:**
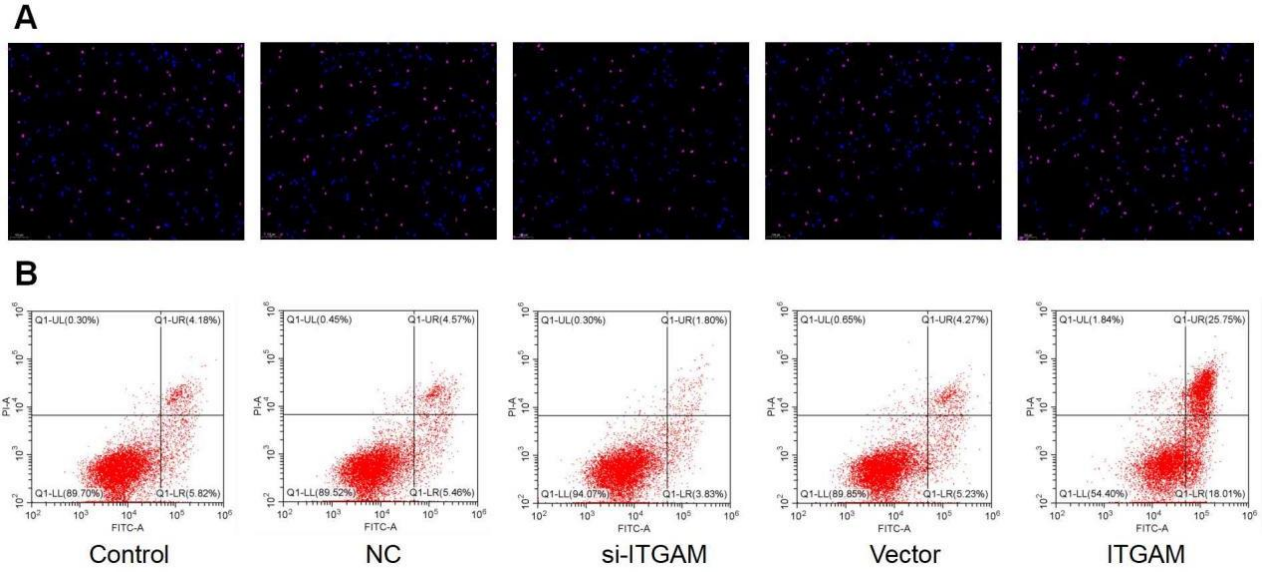
**TUNEL and flow cytometry analysis.** TUNEL analysis of ITGAM in different settings including si-ITGAM, NC, Vector, ITGAM and control. (**A**) TUNEL analysis of ITGAM. (**B**) Flow cytometry analysis.

TUNEL analysis was performed to investigate the impact of ITGAM on neuronal apoptosis. These findings revealed that inhibiting ITGAM with siRNA (si-ITGAM) led to a discernible decrease in neuronal apoptosis. In contrast, overexpression of ITGAM accelerated neuronal apoptotic processes. The specificity of these effects was further affirmed as neither the negative control (NC) nor the Vector groups showed any substantial alterations in neuronal apoptosis (Figure 8A). The above findings were confirmed by flow cytometry analysis to assess the apoptotic cells (Figure 8B). The findings of flow cytometry supported the evidence that ITGAM plays a multifaceted role in ischemic stroke pathophysiology.

## DISCUSSION

Stroke can be divided into IS and hemorrhagic stroke, of which IS accounts for the highest proportion [8]. The procedure of stroke onset has not been explained fully yet and may be associated with age and gender, and is closely linked to hypertension [9]. The IS, as a common clinical disease, can lead to sequelae that pose a huge financial burden and economic stress to patients and their families. Surgical treatment and chemotherapy are common treatment methods for ischemic stroke [10, 11]. Therefore, it is crucial to explore the etiology of IS and its treatment, thus alleviating the suffering of patients. The molecular mechanisms that contribute to the occurrence of the disease, as well as its development, are examined efficiently through microarray and bioinformatics analyses. This knowledge is helpful in the detection of biomarkers that may have potential diagnostic functions and for detecting genetic alterations.

However, in the case of analysis of data as a single microarray dataset, increased rates of false positives and one-sided results were detected. Therefore, two mRNA microarray datasets (GSE16561 and GSE22255) containing 59 IS samples and 44 normal controls were merged. In total, 226 DEGs were screened using the limma package, including 152 up-regulated DEGs and 74 down-regulated DEGs. These DEGs may be potential biomarkers and therapeutic targets for IS. PGAM5 is likely to be a target for the therapy of ischemic stroke [12].

In addition, enrichment analyses showed that changes in the level of expression of these DEGs affect inflammatory immune response-related signal transduction pathways, such as the immune system process, IL-17 signaling pathway, TNFA signaling via NFKB and inflammatory response. The inflammatory immune response has been confirmed to be involved in every stage of IS-related pathological progression [13]. The brain infiltration of T cells is an important process that promotes inflammatory tissue damage after stroke [14]. The T cells play a major immune role in IS, as depicted in an animal model of IS, wherein brain infarct size was decreased in T cell knockout mice but increased after administration of the T cell gene. The T cells adversely affect stroke by promoting the adhesion of leukocytes to the cerebrovascular system and triggering thrombotic inflammation in animals [15]. Furthermore, the AGE/RAGE and MAPK/ERK signaling pathways also play important roles in ischemic stroke [16, 17]. In the present study, ten DEGs were screened as hub genes, and the enrichment analysis results of these hub genes were similar to the enrichment analysis results of all DEGs. Therefore, inflammation and immune response are crucial in IS progression [18]. The process of designing inflammation- and immune response-based targeted interventions may provide novel insights into the treatment of IS. Moreover, our ROC analysis revealed remarkable diagnostic potential for ITGAM, and MMP9 in distinguishing between normal and IS groups.

Finally, multiple key TFs acting on hub genes were predicted by TRRUST database search, including NFKBIA (NF-κB), ARNT, and STAT3. Curcumin significantly reduced the inflammatory response and attenuated brain injury after IS by inhibiting NF-κB phosphorylation [19]. In addition, miRTarBase database search yielded the key miRNAs acting on hub genes, including hsa-miR-338-3p, hsa-miR-5089-3p, and hsa-miR-155-5p, whose down-regulation could attenuate ischemic brain injury [20]. Furthermore, the key drugs acting on hub genes were predicted by the DSigDB database as SB202190, 1,9-Pyrazoloanthrone, and chitosamine (glucosamine). According to a research report, glucosamine acts as a post-ischemic immunomodulator in a sex-dependent manner and may have therapeutic potential in men after stroke [21]. Therefore, these TFs and miRNAs may have an important role in the progression of IS, and the predicted drugs could function as potential agents for the treatment of IS.

To validate the findings of the pharmacological analysis, we performed a series of experiments to determine the MMP9 and ITGAM expression in IS and normal patient groups. The qPCR results revealed that both MMP9 and ITGAM are upregulated in IS samples. This is consistent with existing literature that describes the pro-inflammatory roles of MMP9 and ITGAM in neuroinflammatory diseases [22, 23]. MMP9 is known to degrade extracellular matrix proteins, facilitating the infiltration of inflammatory cells into the brain, a critical step in the exacerbation of IS [22]. Similarly, ITGAM has been implicated in mediating the adhesion and transmigration of leukocytes, which plays a significant role in the inflammatory response to IS [23].

Manipulation of ITGAM expression was also found to regulate the release of key inflammatory cytokines IL-1β, IL-6, and TNF-α, echoing earlier findings on the gene’s role in mediating inflammatory responses [24]. This is particularly interesting given the established role of these cytokines in stroke pathology [25]. Furthermore, ITGAM not only influences inflammation but also affects neuronal apoptosis. Interference with ITGAM inhibited apoptosis, while its overexpression promoted it. These results corroborate the dual role of ITGAM in inflammation and apoptosis. The interplay between ITGAM, inflammation, and apoptosis thus offers a complex yet promising avenue for targeted therapy in IS, requiring a nuanced approach for therapeutic modulation.

The present study also had some limitations as only two IS cohorts were included in this study. Consequently, the small sample size may lead to biased results. In addition, animal models that can validate the underlying mechanisms of IS pathogenesis and the efficacy of potential therapeutic agents are lacking.

## CONCLUSIONS

In conclusion, this study investigated the pathogenesis of IS using bioinformatics and experiments. Inflammation and immune responses are associated with the onset and progression of IS. In addition, ten hub genes and three candidate drugs for IS treatment were identified. The data unearthed in this research provide further insight into the pathogenesis of IS and provide further treatment options and efficient clinical diagnosis for IS.

## MATERIALS AND METHODS

### Data acquisition

The Gene Expression Omnibus (GEO) (http://www.ncbi.nlm.nih.gov/geo/) [26] was utilized to access all the relevant information. The dataset GSE16561 [27], based on the GPL6883 platform, contains peripheral whole blood mRNA expression profiles of 39 IS patients and 24 controls. The GSE22255 [28], per the GPL570 platform, contains peripheral blood mRNA expression profiles from 20 IS patients and 20 healthy controls. The dataset GSE58294 [29], based on the GPL570 platform, contains peripheral whole blood mRNA expression profiles of 69 IS patients and 23 controls. Table 4 displays the specific information of datasets. Subsequently, their gene probes were annotated as gene symbols by their respective platform files. In addition, the gene symbols detected in multiple probes were calculated using their average expression levels. The raw data were log2-transformed and quantile-normalized and batch effects were removed by the ComBat method [30]. Finally, 59 IS samples and 44 healthy control samples were obtained after merging GSE16561 and GSE22255. GSE58294 was used as a validation set.

**Table 4:** Specific information of the dataset.

**Accession**	**Study type**	**Platform**	**Organism**	**Tissue**	**Samples (total, patient/healthy)**
GSE16561	microarray	GPL6883	Homo sapiens	blood	63 (39/24)
GSE22255	microarray	GPL570	Homo sapiens	blood	40 (20/20)
GSE58294	microarray	GPL570	Homo sapiens	blood	92 (69/23)

### Differential expression analysis

The microarray data were screened for differential expression utilizing the linear models constructed per the generalized linear model. This research utilized the R package limma 3.40.6 [31] for the analysis of DEGs in the experimental and control groups. Additionally, we performed multiple linear regression using the lmFit function on the merged dataset and further used the eBays function to compute moderated t-statistics, moderated F-statistic, and log-odds of differential expression by empirical Bayes moderation of the standard errors towards a common value. Afterward, the differential significance of every gene was obtained.

### Enrichment analyses

Gene Ontology (GO) annotation of genes from the R package org.Hs.eg.db [32] (version 3.1.0), gene annotation of the Kyoto Encyclopedia of Genes and Genomes (KEGG) Pathway, and h.all.v7.4.symbols.gmt subset downloaded from the Molecular Signatures Database [33] was used for gene set functional enrichment analyses. Subsequently, genes were mapped to the background set, and the enrichment of gene sets was analyzed through the R package clusterProfiler 3.14.3 [34]. Additionally, the gene sets ranged from 5 to 5000, and the statistically significant values were taken as P < 0.05 and FDR < 0.05.

### Protein-protein interaction network construction

The database STRING (https://string-db.org/) was utilized for constructing a PPI network for DEGs [35], wherein significant interactions were considered to be ones with a combined score greater than 0.7, and the software Cytoscape v.3.7.1 was utilized to identify the hub genes by employing the cytoHubba plugin [36]. Furthermore, functional enrichment analyses of hub genes were carried out by Metascape (http://metascape.org/gp/index.html#/main/step1) [37]. P-value< 0.05 was utilized as a cutoff value.

### Bioinformatics analysis of hub genes

To investigate the relationship between hub genes and clinical characteristics of IS patients, Wilcoxon rank sum tests were utilized. Furthermore, the ROC curve was produced using the ‘pROC’ package and depicted with the ‘ggplot2’ package for better visualization.

The transcription factors (TFs), miRNAs, and small-molecule drugs acting on hub genes were predicted by searching for TRRUST, miRTarBase, and DSigDB databases, respectively, in the Enrichr platform (http://amp.pharm.mssm.edu/Enrichr/) [38].

### Cell culture and treatments

Cortical neuronal cells were retrieved from liquid nitrogen storage and rapidly placed in a 37°C water bath. The cryopreservation tube was gently shaken to dissolve the cryoprotective solution. After complete dissolution, the cells were transferred to a 15 mL centrifuge tube containing 5 mL of DMEM medium enriched with 10% fetal bovine serum (Procell, China). The tube was centrifuged at room temperature at 1000 rpm for 5 minutes. The supernatant was carefully discarded, and the cell pellet was resuspended in a complete culture medium. On reaching the confluence, sub-culturing was performed. The cells were seeded into the culture plates at a density of 5×10^5^ cells per well and incubated overnight at 37°C in a 5% CO2 incubator. Additional experiments involved transfection with siRNA targeting ITGAM (si-ITGAM) to specifically knock down ITGAM gene expression. An empty vector served as a control for these experiments to account for any off-target effects or alterations due to the transfection process itself.

### TUNEL analysis

TUNEL assay was performed by using Cell Apoptosis Detection Kit (Vazyme, China). The neuronal cortical cells were fixed, placed within paraffin, and mounted on glass slides. Immerse slides with adherent cells in 4% paraformaldehyde (pH 7.4) solution at room temperature for 15 minutes, followed by washing three times with PBS, each for 5 minutes. In a succinct overview, the 3’-OH termini of fragmented DNA were enzymatically labeled with digoxigenin-dUTP utilizing Terminal Deoxynucleotidyl Transferase (TdT) as the catalyzing agent. Visualization of apoptotic nuclei was achieved through the application of a rhodamine-conjugated anti-digoxigenin antibody. For nuclear contrast, sections were subsequently counterstained with DAPI. Rinsed the excess DAPI with PBST four times, each for 5 minutes. Slides with a mounting medium containing an anti-fading agent were sealed and observed under a fluorescence microscope.

### Flow cytometry analysis

The cultured cortical neuron cells in the logarithmic growth phase were seeded at 5×10^5^ cells per well in a 6-well culture plate and incubated overnight at 37°C and 5% CO2. To assess apoptotic cells, cells were collected and resuspended in binding buffer. Subsequently, 100 μL of the resulting cell suspension was combined with 5 μL of Annexin-V–FITC (Nanjing KeyGen Biotech, China) and 5 μL of Propidium Iodide (PI). After 15 minutes of incubation period, the cell mixture was further diluted with 300 μL of binding buffer and analyzed using the CytoFLEX Flow cytometer (Beckman, USA).

### RT-PCR

Total RNA from the cortical neurons was isolated using a TRIzol reagent kit (Invitrogen, USA). RNA purity was tested using Nanodrop 2000. Total RNA was reverse transcribed into cDNA using RevertAid First Strand cDNA Synthesis Kit (Thermo Fisher Scientific, USA). Gene expression levels were quantified by Real-Time PCR System (Applied Biosystems, USA). GAPDH was used as a positive internal control, and the mRNA expression levels were calculated according to the 2 − ΔΔCT method. Each sample was analyzed in triplicate. The primer sequences used in this study were as follows: Homo GAPDH (forward: 5’- TCAAGAAGGTGGTGAAGCAGG-3’; reverse: 5’-TCAAAGGTGGAGGAGTGGGT-3’); Homo MMP9 (forward: 5’-AGATGCGTGGAGAGTCGAAA-3’; reverse: 5’-GGTGATGTTGTGGTGGTGC-3’); Homo ITGAM (forward: 5’-GGGAAGTGGCAAGGAATGTA-3’; reverse: 5’-GTCTGTCTGCGTGTGCTGTT-3’); Homo IL-1β (forward: 5’-CGAATCTCCGACCACCACTA-3’; reverse: 5’- AGCCTCGTTATCCCATGTGT -3’); Homo IL-6 (forward: 5’-AGGAGACTTGCCTGGTGAAA-3’; reverse: 5’-CAGGGGTGGTTATTGCATCT-3’); Homo TNF-α (forward: 5’-TCAGAGGGCCTGTACCTCAT-3’; reverse: 5’-GGAAGACCCCTCCCAGATAG -3’.

### Western blot

The harvested cells were subjected to lysis using RIPA buffer fortified with protease and phosphatase inhibitors (G2002, Servicebio, China). Protein concentration was assessed utilizing a bicinchoninic acid (BCA) assay kit (Gbs, G3522-3). Subsequently, protein samples were electrophoresed on 12% SDS-PAGE gels and electrotransferred onto PVDF membranes (Millipore, USA). Blocking was performed with 5% non-fat milk in TBST for two hours at ambient temperature. The membranes were then probed with primary antibodies including si-ITGAM (1:2000, Abcam, UK), ITGAM (1:2000, Sanying Biotechnology, China) and GAPDH (1:1000, Xianzhi Biological, China) and incubated overnight at 4°C. Following primary incubation, membranes were exposed to horseradish peroxidase-conjugated secondary antibodies (Goat Anti-Mouse IgG, Goat Anti-Rabbit IgG, 1:10000, Sanying Biotechnology, China) for an additional hour at room temperature. Protein bands were made visible using ECL detection systems (Servicebio) and were normalized to GAPDH as an internal standard. Densitometric quantification of band intensities was conducted using ImageJ software.

### Statistical analysis

The statistical analyses of the data were performed automatically on the online databases. The code processing of the analysis was done using the R package. All the cell culture analyses were performed using GraphPad prism. The data were expressed as the mean ± standard deviation (SD). Statistical significance in changes between groups was determined using Student’s *t*-test. The value of *p*<0.05 designated an outcome that could be considered significant. All experiments were performed at least three times.

### Availability of data and materials

The data that support the findings of this work are obtainable from GEO (https://www.ncbi.nlm.nih.gov/geo/). GSE22255: https://www.ncbi.nlm.nih.gov/geo/query/acc.cgi?acc=GSE22255. GSE16561: https://www.ncbi.nlm.nih.gov/geo/query/acc.cgi?acc=GSE16561. GSE58294: https://www.ncbi.nlm.nih.gov/geo/query/acc.cgi?acc=GSE58294.

## Supplementary Material

Supplementary Figure 1

Supplementary Table 1
