# Supplementary material for: ITGAM is a critical gene in ischemic stroke
Source: Aging (Albany NY). 2024 Apr 17;16(8):6852–67. doi: 10.18632/aging.205729 (PMC11087101; doi:10.18632/aging.205729)
Supplement: Supplementary Figure 1 [file aging-16-205729-s001.pdf]

SUPPLEMENTARY FIGURE

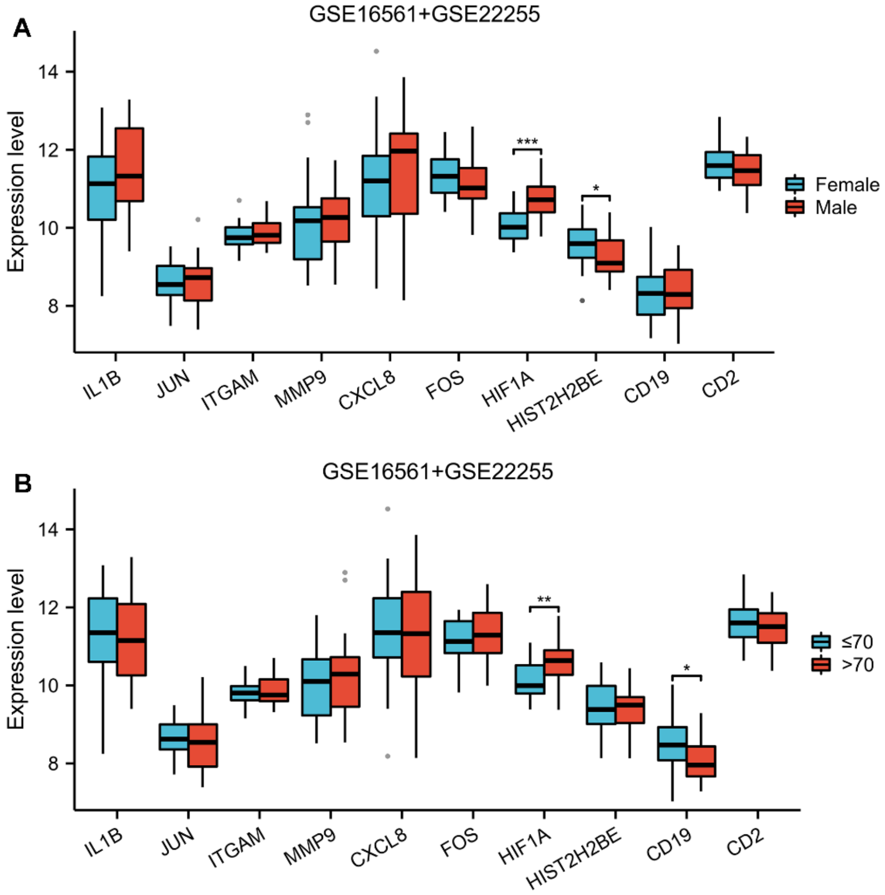

Supplementary Figure 1. Clinical correlation analysis of hub genes. Box plot showing the expression pattern of the hub genes in different genders (male and female, **A**) and ages (≤70 and >70, **B**) of patients in the GSE16561+GSE22255 dataset.
